# *Panax ginseng—Polygonum cuspidatum* is beneficial for alleviating atherosclerosis in ApoE^−/−^ mice by modulating the composition of gut microbiota and related metabolites

**DOI:** 10.3389/fcvm.2026.1773819

**Published:** 2026-04-07

**Authors:** Ya Wang, Jinyi Fu, Jingyi Zhan, Yuanbin Liang, Ruofan Chen, Linjing Su, Qingbing Zhou, Ying Zhang, Weihong Cong, Fengqin Xu

**Affiliations:** 1 Dongzhimen Hospital, Beijing University of Chinese Medicine, Beijing, China; 2 Institute of Geriatrics, Xiyuan Hospital, China Academy of Chinese Medical Sciences, Beijing, China; 3 Cardiovascular Laboratory, Xiyuan Hospital, China Academy of Chinese Medical Sciences, Beijing, China; 4 Key Laboratory for Preventing Vascular Aging by Combination of Disease and Syndrome, National Administration of Traditional Chinese Medicine, Beijing, China

**Keywords:** atherosclerosis, gut microbiota, metabolite, metagenomics, traditional medicine

## Abstract

**Background:**

Atherosclerosis (AS) is a central pathological driver underlying most cardiovascular diseases. Gut microbiota and related metabolites participate in regulating atherosclerosis. *Panax ginseng* and *Polygonum cuspidatum* (GP) herb pair has traditionally been used for cardiovascular diseases. Some active compounds in GP have shown anti-atherosclerotic effects and the effects of GP still needs more evidence-based supports. Therefore, this study aims to investigate the potential effects of GP on atherosclerosis and explore the underlying mechanisms.

**Methods:**

Fifty C57BL/6J ApoE^−/−^ mice were randomly assigned to five groups: model, statin, low-dose GP, medium-dose GP and high-dose GP. They were fed a high-fat diet (HFD) to induce atherosclerosis. Ten wild-type C57BL/6J mice were given chow diet and served as controls. After 12-week intervention, their aortic tissues were collected for Oil Red O staining, colon tissues for Alcian staining and immunofluorescence, and serum samples for measurement of lipid levels and inflammatory cytokines. Then, their fecal DNA was extracted for metagenomic sequencing, while cecum and ileocecal valves were for untargeted metabolomics. Finally, fecal microbiota transplantation was performed to assess the contribution of gut microbiota to observed effects. Twenty additional ApoE^−/−^ mice were randomized to two groups: FMT-Mod and FMT-GPH, given feces from the model or high-dose GP group.

**Results:**

Atherosclerotic plaques accumulated in the aorta and aortic sinus after HFD, while statin and high-dose GP alleviated this burden. TC, TG, LDL-C, MCP-1, MCP-3 and IL-2 showed significant increase after HFD, while statin and GP decreased LDL-C, MCP-1 and MCP-3. The goblet cells, ZO-1 and Occludin decreased after HFD, while statin and GP increased them, indicating that the intestinal barrier integrity was improved. Additionally, the composition of gut microbiota was modulated by GP. Some candidate taxa were identified, such as *Bifidobacteriales*, *Bacteroidetes* and *Escherichia coli*. Twenty-two metabolites were differentially abundant among the control, model and GP groups. Nineteen of them were modulated by HFD and reversed by GP, including 1-methylnicotinamide, dopamine and lysoPA (0:0/18:0). Mice given fecal transplants from the high-dose GP group showed less aortic plaques, lower levels of some lipid and inflammatory cytokines, more goblet cells, more expression of ZO-1 and Occludin, and more 1-methylnicotinamide than those given fecal transplants from the model group.

**Conclusion:**

This study suggests that GP is beneficial for alleviating atherosclerosis in HFD-induced ApoE^−/−^ mice, potentially by modulating the composition of gut microbiota and related metabolites.

## Introduction

Atherosclerosis (AS) is widely considered as an important pathological condition that contributes to the development of various cardiovascular diseases. Dysfunction in the vascular endothelium promotes the accumulation of lipids, fibrous tissue and calcifications, leading to vascular stenosis and triggering inflammation that accelerates atherosclerotic cardiovascular diseases (ASCVDs) (1). Currently, ASCVDs rank among the leading causes of mortality globally, imposing significant health and economic burdens on individuals and society (2, 3). AS and ASCVDs have been regarded as systemic disorders driven by lipid deposition and chronic inflammation. Conventional management of atherosclerosis generally involves lifestyle modification, low-density lipoprotein cholesterol (LDL-C) lowering, and control of blood pressure, glucose and weight (4). Statins are most commonly used, with potential hepatic, renal, and muscle-related adverse effects (5). Traditional medicine, with its multiple targets and multiple pathways (6), shows substantial potential in preventing and treating atherosclerosis.

Recently, gut microbiota and its metabolites have received considerable scholarly attention (7). Gut microbiota is increasingly recognized as a critical interface linking dietary exposures with host metabolism and immune homeostasis. They contribute to influence host health and the development of ASCVDs through the gut-heart axis, by influencing inflammatory responses and lipid metabolism (8). Gut dysbiosis may compromise intestinal barrier integrity, enabling lipopolysaccharide to enter the bloodstream, amplifying inflammatory signaling, and thereby promoting plaque instability and thrombogenesis (9). Among microbiota-derived metabolites, trimethylamine N-oxide is widely regarded as a key molecule associated with atherosclerosis.

As a complementary and alternative medicine, the *Panax ginseng* and *Polygonum cuspidatum* (GP) herb pair has traditionally been used for cardiovascular diseases. In modern researches, ginseng may improve total cholesterol (TC) and LDL-C levels, and improve myocardial energy metabolism remodeling (10–12). Ginsenoside Rg2, an active compound in ginseng, may treat atherosclerosis by anti-inflammation and regulating the phenotypic of vascular cells (13). Ginsenoside Rb1 inhibits the overexpression of inflammatory cytokines, regulates the metabolism of primary bile acids and arachidonic acid in feces, and remodels the gut microbial community (14). Polydatin, an active compound in *Polygonum cuspidatum*, may treat atherosclerosis by anti-inflammation, anti-oxidative stress and regulating lipid metabolism (15). It also may inhibit the activation of NLRP3, decrease interleukins, and improve vascular endothelial function (16, 17). While previous studies have shown that gingseng and polydatin individually alleviate atherosclerosis by remodeling gut microbiota and glycolipid metabolism (18), the effects of GP still require more evidence-based supports.

Based on the hypothesis that GP may modulate atherosclerosis, we conducted an original study to investigate its potential effects in ApoE^−/−^ mice. Given the crucial role of gut microbiota and related metabolites in AS, metagenomic sequencing and untargeted metabolomics were used to explore mechanisms underlying observed effects. To further investigate the role of gut microbiota in the effects of GP, additional mice were subjected to fecal microbiota transplantation (FMT).

## Materials and methods

### *Panax ginseng* and *Polygonum cuspidatum* preparation

Ginseng is the dried rhizomes and roots of *Panax ginseng Meyer*, while *Polygonum cuspidatum* is the dried rhizomes and roots of *Polygonum cuspidatum Sieb. et Zucc*. Herbal medicine samples were prepared and extracted by the pharmacy of Xiyuan Hospital, China Academy of Chinese Medical Sciences (Beijing, China). The processed Chinese herbs were soaked, boiled, filtered, concentrated, and dried into a paste.

The low-dose GP of mice was converted from commonly used clinical doses, corresponding to 10 g/day ginseng and 12 g/day *Polygonum cuspidatum* for a 70-kg adult. Low, medium, and high doses were subsequently set at 1×, 2×, and 4×, respectively. Before use, the extract was removed from a 4 °C refrigerator, dissolved in purified water, and prepared as a suspension of the required concentration.

### Animal and intervention

Male C57BL/6J apolipoprotein E^−/−^ (ApoE^−/−^) mice and wild-type C57BL/6J mice (6 weeks old, 20–25 g) were sourced from Sipeifu Biotechnology Company [license number: SCXK (Beijing) 2019-0010, Beijing, China]. All mice were individually housed in separate cages under specific pathogen-free conditions [license number: SYXK (Beijing) 2019-0051], at 24 ± 2 °C and 55 ± 5% humidity, under a 12-h light/dark cycle.

After 1-week acclimation with normal water and food, ApoE^−/−^ mice (*n* = 50) were maintained on a high-fat diet (HFD; 21% fat, 0.15% cholesterol, and 78.85% standard chow) to induce AS, while C57BL/6J mice (*n* = 10) received chow diet. Following 8 weeks of induction, ApoE^−/−^ mice were randomized into five groups (*n* = 10/group) using a random digit table: model (normal saline i.g), statin (3 mg/kg/day atorvastatin, i.g), GPL (1.3 mg/g/day ginseng and 1.56 mg/g/day *Polygonum cuspidatum*, i.g), GPM (2.6 mg/g/day ginseng and 3.12 mg/g/day *Polygonum cuspidatum*, i.g) and GPH (5.2 mg/g/day ginseng and 6.24 mg/g/day *Polygonum cuspidatum*, i.g). The wild-type mice served as the blank control (equal volume of saline, i.g). The dose of atorvastatin was converted from the adult clinical dose (20 mg/70 kg/day). All treatments were administrated daily through oral gavage (i.g) for 12 weeks.

After the intervention, each mice was transferred to a sterile cage, and their fresh fecal samples without urine were collected in PB buffer (19). The samples were immediately frozen in liquid nitrogen and stored at −80 °C. Then they were fasted and water-deprived for 12 h. Following isoflurane inhalation anesthesia, their blood was collected via retro-orbital sampling, and mice were euthanized by cervical dislocation. Subsequently, their aortic tissues, cecum, and ileocecal valves were collected in separate sterile cryovials and preserved at −80 °C.

### Oil red O staining

The whole aortas (*n* = 3) and 8-µm cross-sections of the aortic sinus (*n* = 3) were stained with Oil Red O solution for 8–10 min, and counterstained with hematoxylin for 3–5 min. Stained specimens were imaged by light microscopy, and plaque area was quantified using Image-Pro Plus 6.0.

### Serum lipid profile and inflammatory cytokines

Blood samples were centrifuged (3,000 r/min, 10 min, 4 °C), and serum was separated. Lipid levels, including LDL-C, high-density lipoprotein cholesterol (HDL-C), TC, and triglycerides (TG), were measured using assay kits (Cat. Nos. A113-1, A112-1, A111-1, A11-1; Nanjing Jiancheng Bioengineering Institute, China). Serum inflammatory cytokines were analyzed using the Mouse Group 1 16-Plex (QuantoBio, Beijing, China), including eotaxin, monocyte chemoattractant protein-1 (MCP-1), MCP-3, interleukin-2 (IL-2), and tumor necrosis factor-alpha (TNF-α).

### Alcian staining

Following graded ethanol dehydration, paraffin infiltration, and embedding, colon tissues (*n* = 3) were sectioned at 4-µm thick. Subsequently, they were deparaffinized and stained with Alcian blue solution A for 10–15 min, followed by solution B for 3 min. Finally, they were dehydrated, coverslipped, and examined by light microscopy.

### Immunofluorescence staining

Paraffin-embedded colon sections were antigen-retrieved in ethylenediaminetetraacetic acid solution (pH 8.0), treated with 3% H_2_O_2_ for 25 min, and blocked for 30 min. Then they were treated with zonula occludens-1 (ZO-1) antibody and incubated overnight at 4 °C, and Cy5-conjugated Affinipure goat anti-rabbit Immunoglobulin G (IgG) at 25 °C for 50 min. They were subsequently incubated with Occludin antibody at 4 °C overnight and AF488-labeled goat anti-rabbit IgG at 25 °C for 50 min. Finally, 4′,6-diamidino-2-phenylindole and an auto-fluorescence quencher were applied, and sections were examined under a fluorescence microscope.

### Metagenomic sequencing analyses

Shotgun metagenomic sequencing was conducted by Beijing QuantiHealth Technology Company (Beijing, China). DNA in 3.5 μL fecal samples was fragmentated, linked to an adaptor, purified and selected. 1μl library, 9.5 μL Dilution Buffer and 9.5 μL mineral oil were mixed and centrifuged for 5 min to detect library length. The library was diluted 40,000-fold with DNA Dilution Buffer (pH 8.0), and constructed using KAPA HyperPlus Kits for qPCR. Then sequenced on the Illumina platform (NovaSeq6000) with three duplicates each sample. Raw sequencing reads underwent quality control using MOCAT2. Metagenomics was assembled using MEGAHIT (1.1.2) and evaluated using QUAST (2.3). Fecal microbiota was classified using MetaPhlAn3 to determine relative species abundance at each taxonomic level.

### Untargeted metabolomic analyses

Cecum and ileocecal valves were dried, ground and centrifuged. The supernatant was subjected for untargeted metabolomic using a Vanquish ultra high-performance liquid chromatography system. Raw metabolomic data were analyzed using ProteoWizard and R, and annotated against the BiotreeDB (V2.1) mass spectrometry database. Differential metabolites were identified using univariate analysis (*p* < 0.05 and |log_2_ fold change| > 0), and multivariate analysis (Variable Importance in Projection score > 1). MetaboAnalyst 6.0 (https://www.metaboanalyst.ca/) was used for pathway enrichment analysis.

### Fecal microbiota transplantation (FMT)

Tweenty additional healthy male C57BL/6J ApoE^−/−^ mice were fed with HFD, and given antibiotics [0.5 g/L vancomycin, 1 g/L ampicillin, 1 g/L metronidazole, and 1 g/L neomycin sulfate (20–22)] for 7 days to deplete their endogenous gut microbiota. These mice were randomized into two groups (*n* = 10 each): FMT-Mod (1 mg/g/3 day model feces transplantation, i.g) and FMT-GPH (1 mg/g/3 day GPH feces transplantation, i.g).

Fresh fecal samples from the model or GPH groups were resuspended in sterile saline and clarified by centrifugation (2,000 r/min, 1 min, 4℃). Mice in the FMT-Mod group were gavaged with the model-derived fecal suspension, while those in the FMT-GPH group received the GPH-derived suspension. Gavage was performed once every 3 days for 12 weeks. The anesthesia, sample collection and detection were performed as previously described. The animal intervention flowchart is shown in Supplementary Figure S1.

### Statistical analysis

Statistical analyses, including one-way ANOVA, Brown-Forsythe and Welch ANOVA and so on, were performed and visualized using GraphPad Prism 9.5.1. Statistically significance was considered as *p* < 0.05.

## Results

### GP alleviated HFD-induced aortic plaque in ApoE^−/−^ mice

**Figure 1:**
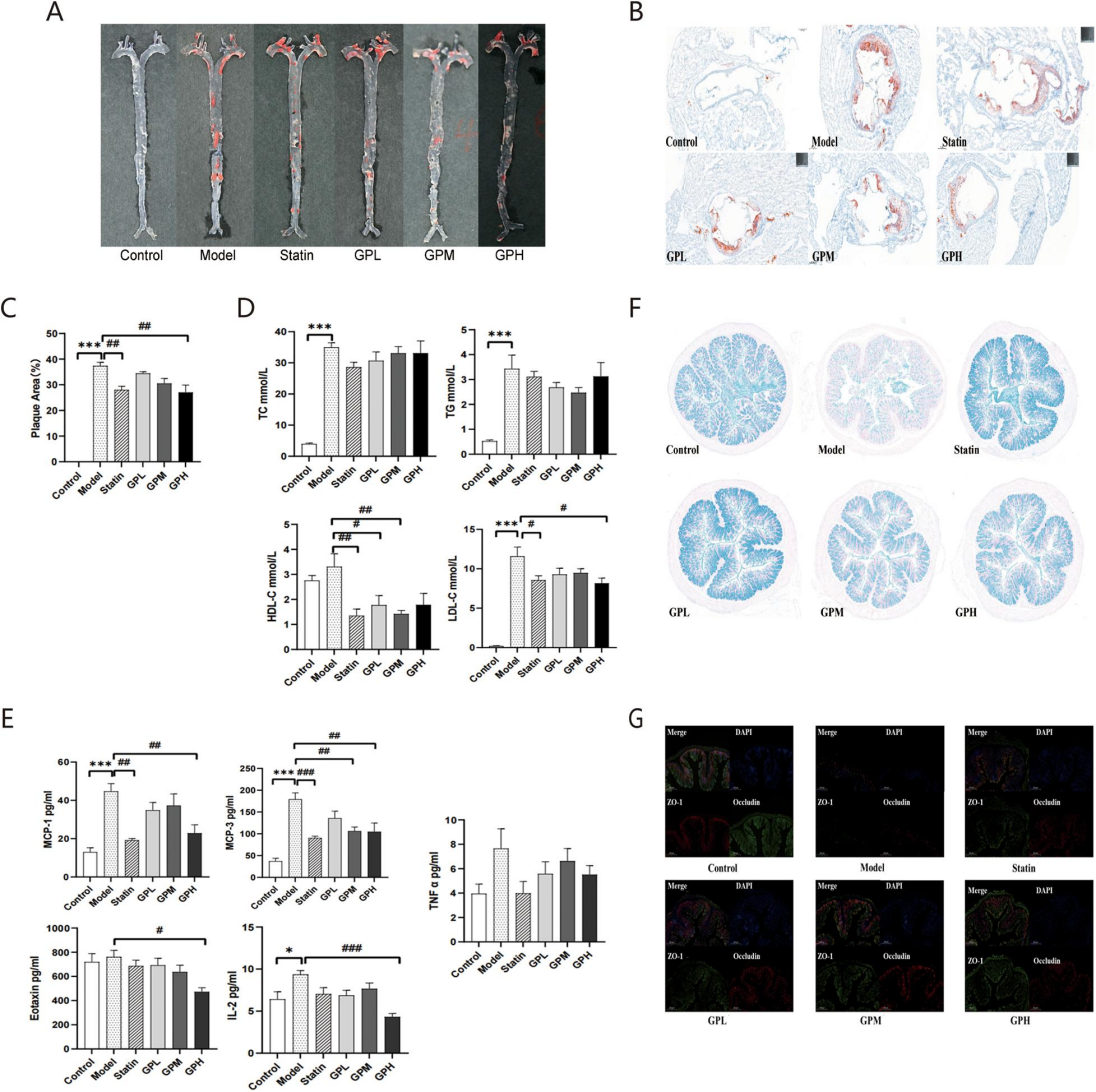
GP reduced HFD-induced plaque formation, reduced levels of lipid and inflammatory cytokines, and maintained the intestinal barrier integrity. **(A)** Representative images of the whole aortas after oil red O staining. **(B)** The aortic sinus. **(C)** Quantification comparison of the plaque area in the aortic sinus. **(D)** Levels of serum lipid profiles (mean with SEM). **(E)** Levels of inflammatory cytokines (mean with SEM). **(F)** Goblet cells in the colon following Alcian blue staining (5X, 200 μm). **(G)** The immunofluorescence staining of ZO-1 and Occludin protein in the colon (5X, 200 μm). *p < 0.05, ***p < 0.001, vs. control. #*p* < 0.05, ##*p* < 0.01, ###*p* < 0.001, vs. model. GP, *Panax ginseng* and *Polygonum cuspidatum*; HFD, high-fat diet; SEM, standard error of the mean.

Figures 1A,B depicts the plaque accumulation in the aorta and aortic sinus of all groups. Plaque burden was higher in the model group than in the control, while it appeared to be lower in the statin and GP groups than in the model group to varying degrees. Figure 1C shows the quantitative comparison of aortic sinus plaque area, presented as the mean percentage of three samples per group. The plaque area was greater in the model group than the control (*p* < 0.001), suggesting that HFD successfully induced atherosclerotic plaques in ApoE^−/−^ mice. Futhermore, it was less in the statin and GPH groups than in the model (*p* < 0.01), indicating that statin and GPH effectively inhibited HFD-induced atherosclerotic progression.

### GP reduced lipid levels and inflammatory cytokines in ApoE^−/−^ mice

After 20-weeks on HFD, serum lipid (Figure 1D) and inflammatory cytokines (Figure 1E) levels were assessed. Significant differences were observed in TC (*p* < 0.001), TG (*p* < 0.001), LDL-C (*p* < 0.001), MCP-1 (*p* < 0.001), MCP-3 (*p* < 0.001), and IL-2 (*p* < 0.05) levels between the model and control groups, suggesting dyslipidemia and inflammatory responses in ApoE^−/−^ mice. Statin significantly reduced HDL-C (*p* < 0.01), LDL-C (*p* < 0.05), MCP-1 (*p* < 0.01) and MCP-3 (*p* < 0.001) levels. Additionally, GPH treatment significantly reduced LDL-C (*p* < 0.05), MCP-1 (*p* < 0.01), MCP-3 (*p* < 0.01), Eotaxin (*p* < 0.05) and IL-2 (*p* < 0.001) levels. GPM reduced HDL-C (*p* < 0.01) and MCP-3 (*p* < 0.01) levels compared with the model group. GPL induced the HDL-C (*p* < 0.05) level. Although some changes were not statistically significant, downward trends in statin and GP groups were observed. Furthermore, the effects of GP on lipid and inflammatory levels did not exhibit a linear correlation with the dose, and GPH showed better effects totally.

### GP maintained intestinal barrier integrity

Figure 1F illustrates goblet cells in the colon, while Figure 1G shows colonic ZO-1 and Occludin protein expression. They are integral components of the intestinal barrier, served as indicators of the intestinal barrier integrity. They were lower in the model group than in the control, suggesting that HFD may be harmful for the intestinal barrier in ApoE^−/−^ mice. They were more in the statin and GP groups than in the model, indicating that statin and GP may reverse influences of HFD on intestinal barrier and maintain its integrity.

### GP modulated gut microbiota composition

**Figure 2:**
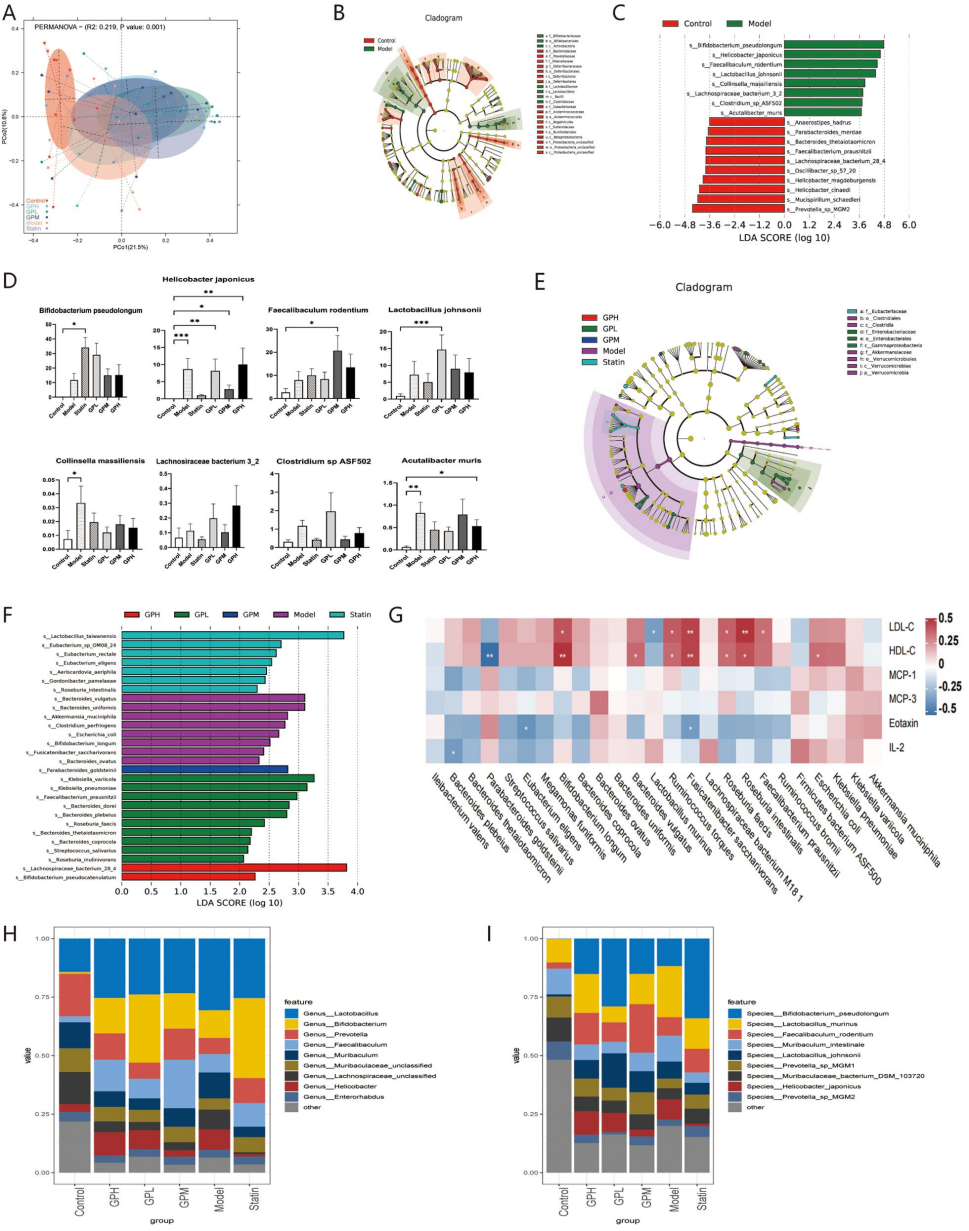
The composition of gut microbiota was modulated by HFD, statin and GP. **(A)** PCoA analysis based on the Bray-Curtis distance matrix. **(B)** The cladogram between the control and model groups in LEfSe analysis. **(C)** Species with the top LDA scores in the control and model groups. **(D)** The abundance comparison of gut microbiota which was significantly enriched in the model group in **(C)**. **(E)** The cladogram without the control group in LEfSe analysis. **(F)** Species with the highest LDA scores without the control group. **(G)** A heatmap of the correlation between microbiota and lipid or inflammatory cytokines. Blue regions indicate negative correlation, while red regions denote positive correlations. **(H)** Abundances of main genus. **(I)** Abundances of main species. *p < 0.05, **p < 0.01, ***p < 0.001, vs. control. HFD, high-fat diet; GP, *Panax ginseng* and *Polygonum cuspidatum*; PCoA, principal coordinates analysis; LEfSe, linear discriminant analysis effect size; LDA, linear discriminant analysis; SEM, standard error of the mean.

A Bray–Curtis dissimilarity matrix was constructed to quantify β-diversity, followed by principal coordinates analysis (Figure 2A). The variance in community composition was 32.3% (PCo1: 21.5%; PCo2: 10.8%) and points from different groups occupied distinct regions, indicating the differentiation in overall gut microbial community composition. Concordantly, PERMANOVA confirmed a significant difference in β-diversity across groups (Bray–Curtis distance: *R*^2^ = 0.219; *p* < 0.01).

Figures 2B–F depict the linear discriminant analysis effect size in different groups. In the comparison between the model and control groups (Figure 2B), *Bifidobacteriales*, *Lacrobacillales* and *Clostridiaceae* were significantly enriched in the model group (*p* < 0.05), which can be considered as the characteristics of AS. Figure 2C depicts the microbiota with log_10_(LDA) score > 3.6, and Figure 2D shows the comparison of their abundances in all groups. There were no significant differences between the model and treatment groups. Only *Collinsella massiliensis* exhibited opposing trends in the statin and all GP-treated groups compared to it in the model. Figure 2E depicts the cladogram between the model and treatment groups. *Clostridia* class was most abundant in the model group, and *Gammaproteobacteria* class was most abundant in the GPL. Figure 2F presents microbial taxa with log_10_(LDA) scores > 2.0, indicating the microbiota which contributes most strongly to group discrimination. In Figure 2F, *Eubacterium* contributed most in the statin group, *Bacteroides* contributed most in the GPL, *Parabacteroides goldsteinii* contributed most in the GPM, and *Lachnospiraceae bacterium 28_4* contributed most in the GPH.

Table 1 shows the species whose abundances were differential between the model and GP groups (*p* < 0.05). Only *Escherichia coli* was less in all GP doses than in the model group. Figure 2G shows the Spearman's correlation between species in Table 1 and levels of cholesterol or inflammatory cytokines, which are associated with atherosclerotic risks. *Parabacteroides goldsteinii* was negatively correlated with HDL-C (*p* < 0.01), while *Bifidobacterium longum*, *Fusicatenibacter saccharivorans*, and *Roseburia intestinalis* were positively correlated with HDL-C and LDL-C (*p* < 0.05).

**Table 1:** Species with statistically different abundances (*p* < 0.05).

Gut microbiota	GPL vs. model	Gut microbiota	GPM vs. model	Gut microbiota	GPH vs. model
*Bacteroides plebeius*	↑	*Akkermansia muciniphila*	↓	*Escherichia coli*	↓
*Bacteroides uniformis*	↓	*Klebsiella pneumoniae*	↓	*Bacteroides uniformis*	↓
*Lactobacillus murinus*	↓	*Faecalibacterium prausnitzii*	↓	*Ileibacterium valens*	↑
*Bifidobacterium longum*	↓	*Lachnospiraceae bacterium M18 1*	↓	*Bacteroides ovatus*	↓
*Bacteroides coprocola*	↓	*Roseburia faecis*	↓	*Fusicatenibacter saccharivorans*	↓
*Bacteroides thetaiotaomicron*	↑	*Escherichia coli*	↓	*Roseburia intestinalis*	↓
*Streptococcus salivarius*	↑	*Klebsiella variicola*	↓		
*Eubacterium eligens*	↑	*Bacteroides vulgatus*	↓		
*Ruminococcus torques*	↓	*Ileibacterium valens*	↑		
*Megamonas funiformis*	↑	*Bifidobacterium longum*	↓		
*Escherichia coli*	↓	*Bacteroides ovatus*	↓		
		*Fusicatenibacter saccharivorans*	↓		
		*Roseburia intestinalis*	↓		
		*Ruminococcus bromii*	↓		
		*Parabacteroides goldsteinii*	↑		
		*Firmicutes bacterium ASF500*	↓		

Figures 2H,I depicts the species classification using MetaPhlAn3 and their abundances. The microbial community composition differed across groups. At the genus level (Figure 2H), abundances of *Muribaculaceae unclassified* and *Prevotella* (both within the phylum *Bacteroidetes*) were reduced in the model group and appeared to increase with statin or GP treatment, while *Muribaculum* showed an opposite tendency. Additionally, abundances of *Bifidobacterium* were higher in the model group and further increased in statin and GP groups. At the species level (Figure 2I), the relative abundance of *Prevotella sp MGM1* was decreased in the model but increased in the statin and GP groups, while *Muribaculum intestinale* exhibited an opposite trend.

### GP modulated the metabolites in the cecum and ileocecal valves

**Figure 3:**
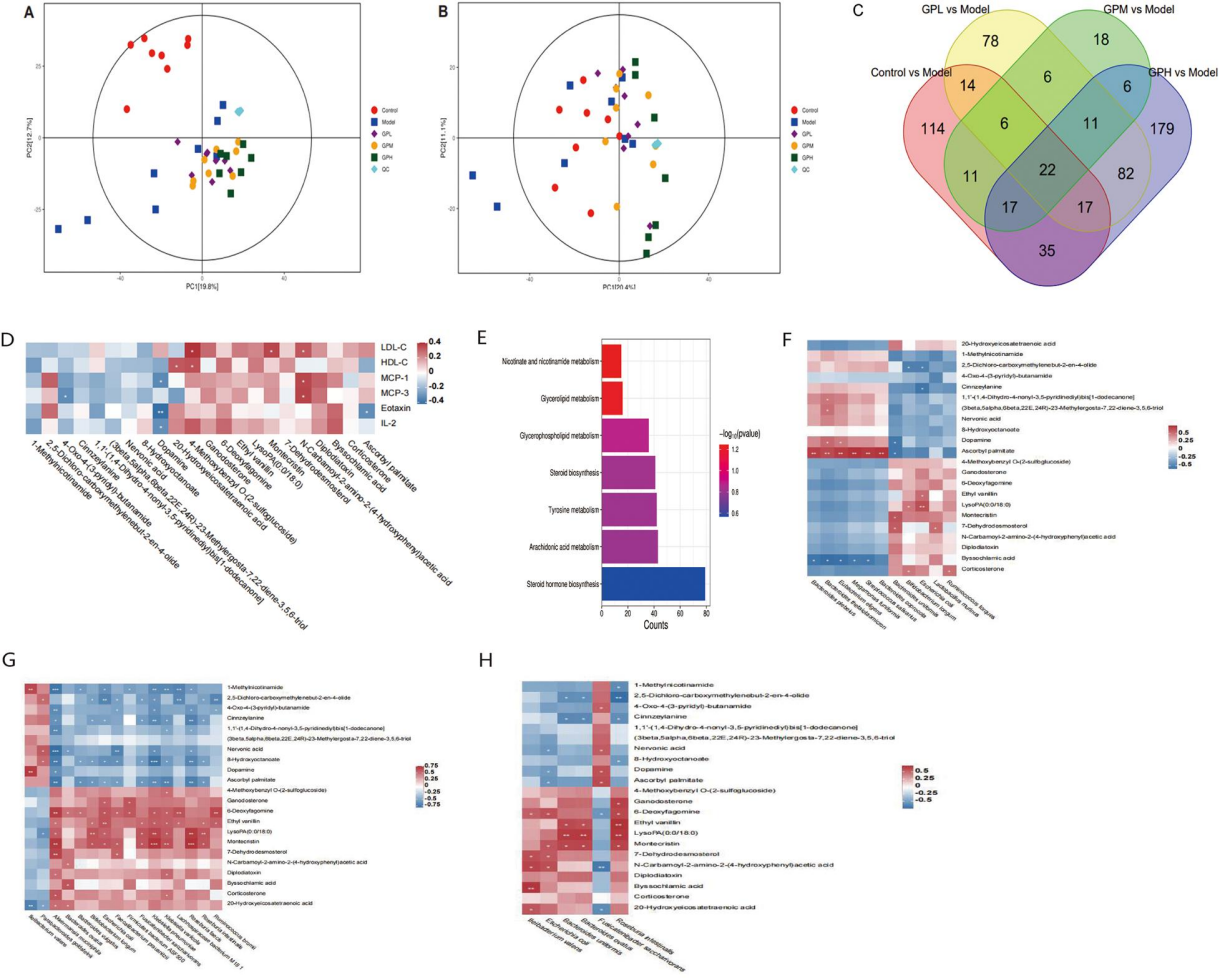
The metabolites in the cecum and ileocecal valves were modulated by HFD and GP. **(A)** PCA score plots in the positive ion mode. **(B)** PCA score plots in the negative ion mode. **(C)** Venn diagram of the numbers of differentially abundant metabolites between the groups. **(D)** The correlation between differentially abundant metabolites and lipids or inflammatory cytokines. **(E)** Pathway enrichment analysis based on Kyoto Encyclopedia of Genes and Genomes. **(F–H)** Correlation between differently abundant species and metabolites in GPL **(F)**, GPM **(G)** and GPH **(H)**. *p < 0.05, **p < 0.01, ***p < 0.001. HFD, high-fat diet; PCA, principal component analysis; GP, *Panax ginseng* and *Polygonum cuspidatum*; GPL, low-dose GP; GPM, medium-dose GP; GPH, high-dose GP.

Figures 3A,B depict score plots in the principal component analysis of metabolites in the cecum and ileocecal valves, to comprehensively characterize the global metabolic profiles across groups. In the plots of positive ion mode (Figure 3A), PC1 and PC2 respectively explained 19.8% and 12.7% of the total variance. In the plots of negative ion mode (Figure 3B), PC1 and PC2 respectively explained 20.4% and 11.1% of the total variance. Figure 3C shows a Venn diagram summarizing the number of metabolites which were differentially abundant between the two groups. Twenty-two metabolites differed (*p* < 0.05) among the model, control and GP groups, whose names and trends were listed in Table 2. Among these, nineteen metabolites were modulated by HFD and exhibited reverse trends in GP groups.

**Table 2:** Core differentially expressed metabolites and their trends.

No.	Differential metabolites	Model vs. control	GPL vs. model	GPM vs. model	GPH vs. model
1	4-Methoxybenzyl O-(2-sulfoglucoside)	↑	↓	↓	↓
2	6-Deoxyfagomine	↑	↓	↓	↓
3	7-Dehydrodesmosterol	↑	↓	↓	↓
4	Byssochlamic acid	↑	↓	↓	↓
5	Diplodiatoxin	↑	↓	↓	↓
6	Ethyl vanillin	↑	↓	↓	↓
7	Ganodosterone	↑	↓	↓	↓
8	LysoPA(0:0/18:0)	↑	↓	↓	↓
9	Montecristin	↑	↓	↓	↓
10	N-Carbamoyl-2-amino-2-(4-hydroxyphenyl)acetic acid	↑	↓	↓	↓
11	(3beta,5alpha,6beta,22E,24R)-23-Methylergosta-7,22-diene-3,5,6-triol	↓	↑	↑	↑
12	1,1′-(1,4-Dihydro-4-nonyl-3,5-pyridinediyl)bis[1-dodecanone]	↓	↑	↑	↑
13	1-Methylnicotinamide	↓	↑	↑	↑
14	2,5-Dichloro-carboxymethylenebut-2-en-4-olide	↓	↑	↑	↑
15	4-Oxo-4-(3-pyridyl)-butanamide	↓	↑	↑	↑
16	8-Hydroxyoctanoate	↓	↑	↑	↑
17	Cinnzeylanine	↓	↑	↑	↑
18	Dopamine	↓	↑	↑	↑
19	Nervonic acid	↓	↑	↑	↑
20	Ascorbyl palmitate	↑	↑	↑	↑
21	Corticosterone	↑	↓	↑	↑
22	20-Hydroxyeicosatetraenoic acid	↓	↓	↓	↓

Figure 3D depicts the Spearman's correlation analysis between metabolites in Table 2 and levels of lipids or inflammatory cytokines. Dopamine was negatively correlated with Eotaxin (*p* < 0.01), MCP-1 (*p* < 0.05), and IL-2 (*p* < 0.05) levels, while it was more abundant in GP groups than the model group. Figure 3E and Table 3 shows the pathway enrichment analysis of the metabolites in Table 2. Seven pathways were enriched and they all *p* > 0.05. 1-Methylnicotinamide was mapped to the nicotinate and nicotinamide metabolism pathway (*p* > 0.05), and dopamine was mapped to the tyrosine metabolism pathway (*p* > 0.05).

**Table 3:** Enrichment pathways.

No.	Pathway	Total	Expected	*p* value	Holm adjust	FDR	Impact
1	Nicotinate and nicotinamide metabolism	15	0.05829	0.056983	1	1	0.1382
2	Glycerolipid metabolism	16	0.062176	0.060683	1	1	0.0125
3	Glycerophospholipid metabolism	36	0.1399	0.13219	1	1	0.139
4	Steroid biosynthesis	41	0.15933	0.14934	1	1	0
5	Tyrosine metabolism	42	0.16321	0.15274	1	1	0.1297
6	Arachidonic acid metabolism	43	0.1671	0.15612	1	1	0
7	Steroid hormone biosynthesis	79	0.30699	0.27069	1	1	0.0068

The associations between species (Table 1) and metabolites (Table 2) were assessed within each group using Spearman's correlation analysis. In the GPL group (Figure 3F), ascorbyl palmitate and byssochlamic acid were correlated with most species (*p* < 0.05) whose abundances was different between the GPL and model groups. In the GPM group (Figure 3G), 1-methylnicotinamide and ascorbyl palmitate were negatively correlated with *Escherichia coli* (*p* < 0.05), and most other species. In the GPH group (Figure 3H), lysoPA (0:0/18:0) was positively correlated with *Roseburia intestinalis* (*p* < 0.05).

### FMT from the GPH group also alleviated atherosclerotic burdens

**Figure 4:**
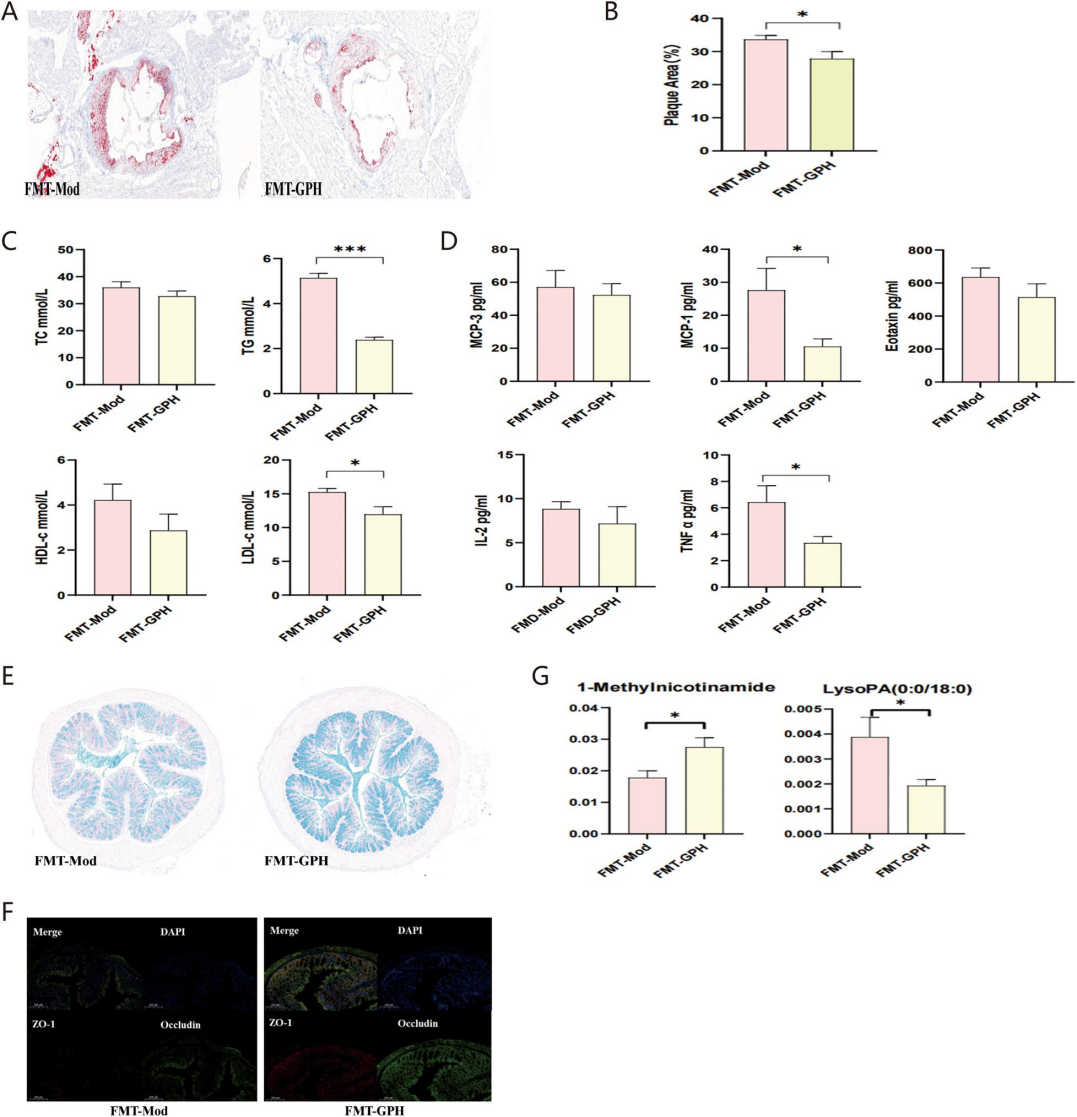
ApoE^−/−^ mice in the FMT-GPH group exhibited fewer atherosclerotic plaques, lower lipid and inflammatory levels, more intact intestinal barrier, and metabolic changes compared with those in the FMT-Mod group. **(A)** Aortic sinus plaque area in FMT groups. **(B)** Statistical comparison of aortic sinus plaques. **(C)** Serum lipid levels. **(D)** Inflammatory cytokine levels. **(E)** Goblet cells in colon tissues after Alcian blue staining (5X, 200 μm). **(F)** Colon tight junction proteins (5X, 200 μm). **(G)** Statistical comparison of main metabolites. *p < 0.05, ***p < 0.001. FMT, fecal microbiota transplantation; GPH, high-dose GP.

Figure 4A shows atherosclerotic plaques in the aortic sinus of the two groups, and Figure 4B provides a quantitative comparison, indicating that mice in the FMT-GPH group exhibited less plaques compared with those in the FMT-Mod group (*p* < 0.05). Additionally, the FMT-GPH group exhibited lower levels of cholesterol (Figure 4C) and inflammatory cytokine (Figure 4D) than the FMT-Mod group, with significant differences in TG (*p* < 0.001), LDL-C (*p* < 0.05), MCP-1 (*p* < 0.05) and TNF-α (*p* < 0.05). Moreover, more goblet cells (Figure 4E) and higher expression of ZO-1 and Occludin (Figure 4F) were observed in the FMT-GPH group, indicating more intact intestinal barriers. Notably, the level of 1-methylnicotinamide was higher in the FMT-GPH group (Figure 4G).

## Discussion

In this study, we investigated the effects of GP on AS in the animal model and further assessed the contribution of gut microbiota through FMT. This study provides evidence to support clinical application of GP for atherosclerosis and highlights several potential microbial taxa as promising targets. We have observed that GP prevented HFD-induced aortic plaque formation, improved lipid profiles and inflammation, preserved intestinal barrier, and modulated gut microbiota composition and relevant metabolites in ApoE^−/−^ mice. FMT showed that feces from the GPH group exerted the similar effects as GP.

Atherosclerotic plaques primarily developed in the aortic arch and arterial bifurcation of HFD-induced ApoE^−/−^ mice, as the key pathological features in AS (23). Across three dose levels, GP was associated with a reduction in aortic plaques, suggesting that GP appeared to slow the progression of AS. Dyslipidemia is a major driver of atherosclerotic initiation and progression, and reducing LDL-C is a primary goal in the secondary prevention of ASCVDs (24). LDL-C accumulates beneath the endothelial layer, promotes oxidative stress, participates in immune responses, and amplifies inflammation, thereby driving atherosclerotic plaque formation (25). In this study, GP not only reduced LDL-C, but also improved inflammation in a dose-independent manner. This dose-response relationship may be associated with the plateau effects, absorption saturation, component interactions or toxic effects. Further studies on pharmacokinetics and pharmacodynamics should be conducted in the future and an expanded sample size should be employed to reduce individual variability. MCP-1 promotes AS by recruiting monocytes and macrophages to the arterial intima and media (26, 27). *In vitro* experiments reveal that MCP-3 promotes the proliferation of human coronary smooth muscle cells (28), which accelerates AS and its complications (29).

Occludin and ZO-1 are intestinal tight junction proteins, while goblet cells represent a kind of specialized intestinal epithelial cells. Along with the mucus produced by goblet cells, they all contribute to the intestinal barrier. Previous studies have also observed that tight-junction proteins are reduced and intestinal permeability is increased in HFD-induced ApoE^−/−^ mice (30, 31), allowing enhanced cholesterol absorption and lipopolysaccharide to enter the bloodstream, which in turn triggers systemic inflammation and immune disorders (32). As GP improved the intestinal barrier permeability in this study, its effects may be associated with the gut-vascular axis.

The gut microbiota also contributes to intestinal barrier homeostasis through colonization resistance, microbial metabolites and immune modulation. It plays an important role in modulating interactions between exogenous factors and host immune system (33). In this study, the gut microbial composition differed across groups. Microbial taxa within the phylum *Actinomycetota, Bacteroidetes* and *Proteobacteria* contributed more to the effects of GP. However, changes of individual species were not significant and some species may exert bidirectional regulatory effects in the host. Accordingly, this study identified several potential microbial taxa, and their individual contributions could be further investigated through functional colonization or mono-association experiments in future studies.

*Bifidobacteriales* (phylum *Actinomycetota*) may have the potential to alter cholesterol metabolism, reduce trimethylamine N-oxide, improve inflammation and inhibit oxidative stress (34). Trimethylamine N-oxide may increase foam cell formation and platelet hyper-reactivity, activate inflammatory responses and endothelial dysfunction, and disturb cholesterol transportation (35). *Bifidobacterium* may be a negative regulator of lipids and glucose, while plays an anti-oxidant role by trapping metal ions and activating relevant enzyme system (36). It inhibits the production of trimethylamine N-oxide and regulate bile acid metabolism (37, 38). Zhao et al. observed that *Collinsella massiliensis* (phylum *Actinomycetota*) was more abundant in the patients of coronary artery disease than people without the disease (39).

*Bacteroidetes* are associated with the production of short-chain fatty acids, and the degradation of intestinal cholesterol (40), protecting the cardiovascular system (41). For instance, acetate inhibits endothelial-to-mesenchymal transition by modulating the TGF-β signaling pathway (42). *Bacteroides ovatus* may alleviate AS by enhancing bile acid metabolism, restoring the M1/M2 polarisation balance and reducing inflammation (43). *Prevotella spp.* is associated with the arachidonic acid metabolism pathway (44). *Prevotella copri* is positively correlated with trimethylamine and bile acids (45, 46). Wu et al. observed that *Muribaculaceae* was negatively associated with inflammatory cytokine levels and aortic plaque burden in HFD-induced ApoE^−/−^ mice (47), regulating the metabolism of propionate and caproate (48). *Parabacteroides goldsteinii* (phylum *Bacteroidetes*) is negatively correlated with HDL-C; its abundance is suppressed by aspirin (49), but increases in the GPM.

Bacterial DNA is present in atherosclerotic plaques, with *Proteobacteria* accounting for 48.3% of the detected sequences (50). *Escherichia coli* (phylum *Proteobacteria*) is one of the major bacteria producers of lipopolysaccharide (51), which can exacerbate AS by promoting inflammatory responses and enhancing oxidative stress. *Escherichia coli*-derived lipopolysaccharide could induce endotoxemia and aortic plaques in ApoE mice (52). You Xi and Gao Bo (53) have observed that *Escherichia coli* was more abundant in patients with coronary heart diseases than in healthy volunteers.

Most of the differentially abundant metabolites in this study have been rarely reported in previous studies, but compounds with similar structures have been reported to exhibit anti-atherosclerotic activities. 1-Methylnicotinamide exhibits anti-AS effects in ApoE/LDLR^−/−/−^ mice, potentially through mechanisms involving reduced inflammation, inhibition of platelet activation, and improved endothelial function (54). Additionally, the N1-methylnicotinamide level is correlated with the presence and progression of ASCVDs (55). LysoPA(0:0/18:0) is a type of lysophosphatidic acid, which is positively associated with lipoprotein(a) levels (56). Lysophosphatidic acid has been observed to be associated with dyslipidemia and aortic AS (57), potentially promotes cell proliferation of vascular cells and influence intestinal barrier integrity (58). The gut microbiota may suggest the capacity to influence dopamine metabolism (59), while dopaminergic signaling may, in turn, modulate key processes in AS, including immune–inflammatory phenotypes and endothelial oxidative stress (60). The absence of statistical significance in all the enriched pathways may be related to factors such as sample size, metabolite interactions, limitations of the enrichment method, and the multi-target effects of GP on the microbiota. Future studies utilizing functional colonization may reveal more effective pathways.

FMT is a therapeutic strategy which has been recommended for recurrent *Clostridioides difficile* infection, while its potential applications in irritable bowel syndrome and inflammatory bowel disease are exploring (61). In this study, FMT was used to explore whether fecal microbiota from GP-treated mice exert effects in ApoE^−/−^ mice. The mice in FMT-GPH group showed ameliorated atherosclerotic manifestations, suggesting that the gut microbiota modulated by GP may also be beneficial for alleviating AS. Several potential microbial taxa have been identified in this study and may represent promising targets for fecal microbiota-based therapeutic strategies in AS.

## Conclusion

This study indicates that GP may help alleviate atherosclerosis in HFD-induced ApoE^−/−^ mice, potentially by reshaping gut microbial community structure and related metabolite profiles. Further validation is required for clinical application, including pharmacokinetic and pharmacodynamic studies, toxicological evaluations, and assessments of efficacy and safety in humans. Gut microbiota may represent an important mediator of the effects observed with GP and promising targets for atherosclerosis. This study identifies several differential microbiota, such as *Bifidobacteriales*, *Collinsella massiliensis*, *Bacteroidetes*, and *Escherichia coli*. Their individual roles should be validated through functional colonization in future studies.

## Data Availability

The datasets presented in this study can be found in online repositories. The names of the repository/repositories and accession number(s) can be found below: https://ngdc.cncb.ac.cn/omix, OMIX014704; https://ngdc.cncb.ac.cn/gsa/, CRA036945.

## Supplementary material

Supplementary Figure S1 Animal intervention flowchart.
