# Supplementary figures and images for: Panax ginseng—Polygonum cuspidatum is beneficial for alleviating atherosclerosis in ApoE−/− mice by modulating the composition of gut microbiota and related metabolites
Source: Front Cardiovasc Med. 2026 Apr 7;13:1773819. doi: 10.3389/fcvm.2026.1773819 (PMC13095641; doi:10.3389/fcvm.2026.1773819)

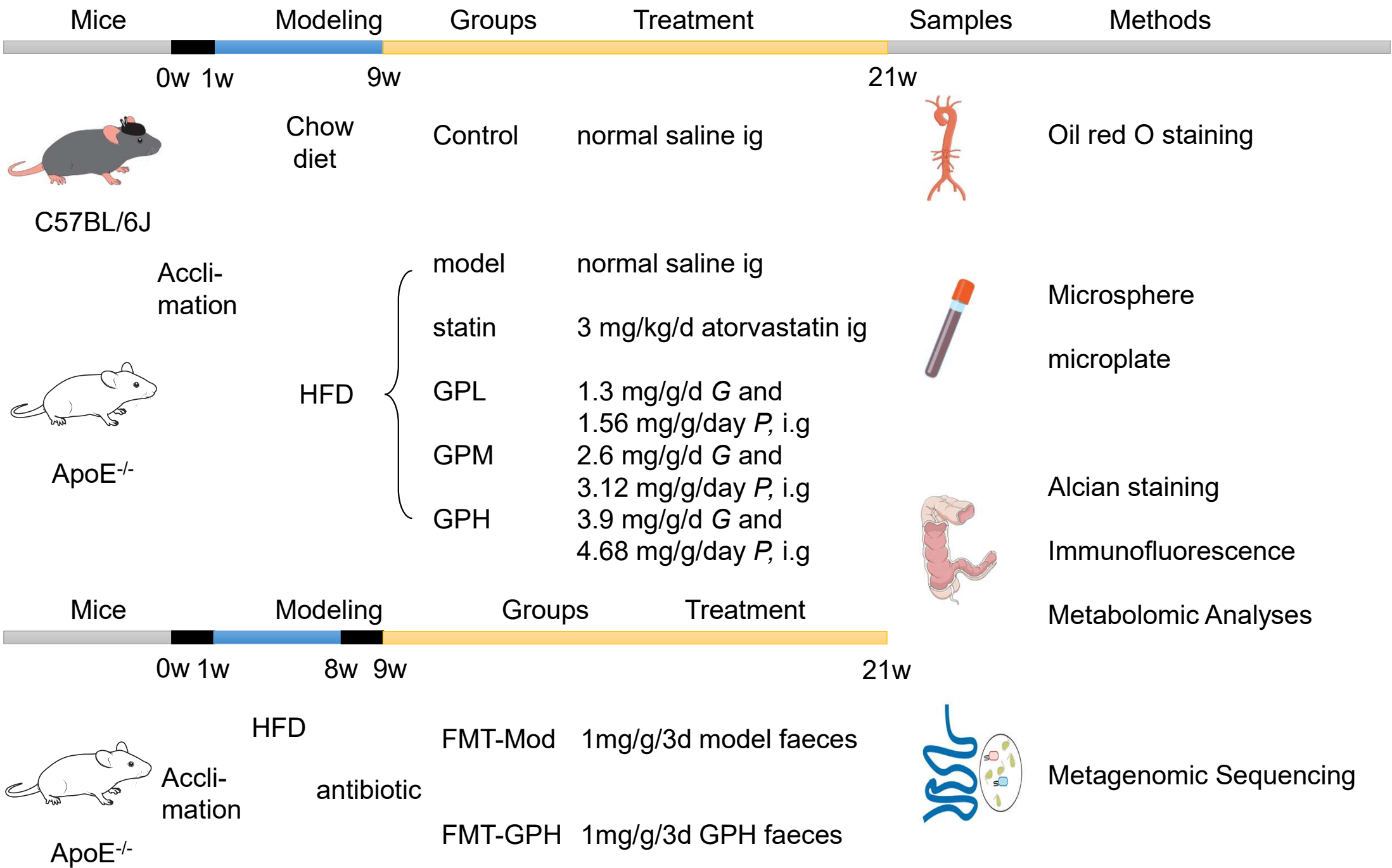

Supplement: Supplementary Figure S1 — Animal intervention flowchart. [file Datasheet1.pdf]
